# Edible Oils Attenuate Button Battery-Induced Injury in Porcine Esophageal Segments

**DOI:** 10.3389/fped.2020.00097

**Published:** 2020-03-13

**Authors:** Wenyuan Jia, Bin Zhang, Guanghui Xu, Jiangang Xie, Haidong Wei, Niqi Shan, Qianmei Wang, Wen Yin, Wei Zhao

**Affiliations:** ^1^Department of Emergency, Xijing Hospital, Air Force Medical University, Xi'an, China; ^2^Division of Digestive Surgery, Xijing Hospital of Digestive Diseases, Air Force Medical University, Xi'an, China; ^3^Department of Anesthesiology, The Second Affiliated Hospital of Xi'an Jiaotong University, Xi'an, China

**Keywords:** button battery ingestion, foreign body, esophageal injury, edible oils, porcine

## Abstract

**Objective:** The objective of the study is to test whether the use of edible oil might be an early treatment strategy for reducing button battery-induced esophageal injury.

**Methods:** A button battery was inserted into esophageal segments collected from pigs. The esophageal segments were randomly allotted to one of the following six treatments: (1) untreated (nothing injected), (2) lemon juice, (3) orange juice, (4) colza oil, (5) peanut oil, and (6) olive oil. Every hour, the battery discharge and the pH value were measured in the esophageal tissue. After treatment for 6 h, the residual voltage of the battery was measured and the esophageal tissue was processed with H&E staining.

**Results:** In esophageal segments of the untreated group, a large area of the mucous membrane was severely eroded. Partial erosion was observed in esophageal tissues treated with either lemon juice or orange juice. Furthermore, the esophageal tissues were basically intact, had little damage when treated with oils. The highest extra-esophageal discharge voltage was recorded in the untreated group, a medium amount of discharge voltage was recorded in the lemon juice and orange juice groups, and the lowest discharge voltage was recorded in all the edible oils groups.

**Conclusions:** Edible oils immersed the battery, reduced the surrounding electrolysis, and thus attenuated battery discharge. As a result, treatment with edible oils attenuated the pH alkalization and tissue damage in button battery injury of pig esophageal segments. These results indicate that edible oils might be used in the treatment of button battery ingestion.

## Introduction

The harmfulness of accidental button battery ingestion on human beings has been documented as early as the 1970s (1). Since then, the frequency of using button batteries has been dramatically increased, and the button battery-induced esophageal injury is increasing (2). According to the National Capital Poison Center (https://www.poison.org/battery/stats), the incidence of accidental button battery ingestion in the United States was 3,240 cases (9.85 per million population) in 2017, of which 4.44% suffered moderate-to-severe esophageal damage, including esophageal perforation, esophagotracheal fistula, large arterio-esophageal fistula, and even death (3). Although the National Capital Poison Center has provided guidelines for the triage and treatment of button battery ingestions (4) and a National Button Battery Task Force established in the U.S. to prevent button battery-induced human injury (5), the incidence of battery ingestions remains high and life-threatening complications are a global problem (6, 7).

Fast removal has been proposed as the golden rule for treating button battery ingestion. However, one should note that the button battery can cause visible esophageal mucosal injury as early as 15 min after ingestion and result in significant mucosal injury within 2 h (8). Unfortunately, there may be multiple delays from the time of ingestion until endoscopic removal of the battery, often as taking as long as 6 h, and unwitnessed ingestions may not be recognized for days, weeks, or even months (9, 10). In this sense, esophageal injury continuously occurs before the button battery is removed in most cases. So, early treatment strategies need to be developed to minimize esophageal injury before battery removal (4).

The underlying mechanisms of button battery-induced esophageal injury have been proposed in recent studies. Briefly, soon after ingestion, battery discharge produces a large number of hydroxide ions (OH-) at the negative electrode, creates an alkaline environment that erodes the esophagus, and finally induces serious consequences such as esophageal perforation. It has been reported that the application of weakly acidic solutions to neutralize alkaline may partly reduce or delay the progression of battery-induced esophageal injury (8). And thanks to the prior work of Anfang et al. where honey and carafate were proposed to create the weakly acidic environment around negative electrode (11), we theorized that methods to create a less or non-electrolytic environment to attenuate or block battery discharge as early as possible may prevent or attenuate the persistent production of OH- at the negative electrode and thereby create a longer window for battery removal procedures. Edible oils are natural, safe, non-electrolyte substances with no electrical conductivity. In this study, we investigated the potential use of edible oils to attenuate button battery-induced esophageal injury in pig esophageal tissues *in vitro*.

## Materials and Methods

### Materials

Esophageal samples were collected from 16 healthy male domestic pigs (Landrace, aged 3–4 months, weighing 24–27 kg) within 5 min after their euthanization at the Abattoir of Wei River Bridge (Shannxi, China). The upper 15-cm of the esophagus was cut into 5-cm-long segments. A total of 48 segments were adopted. Other materials/instruments used in this study included the following: button battery (Panasonic, CR2032, 20 mm in diameter, 3.2 mm in height, 3.0 V), lemon juice (Sunquick, Guangdong, China), orange juice (Nongfu Spring, Zhejiang, China), colza oil (Luhua, Shandong, China), peanut oil (Luhua, Shandong, China), olive oil (Tianyu, Shandong, China), voltmeter (Props Kitt MT-1509-Cn, Taiwan, China), and universal pH indicator paper.

### Establishment of the Button Battery-Induced Esophageal Injury Model

In the first part of the experiment, to mimic the position of the esophagus during human standing, the esophageal segments were positioned with their long axis perpendicular to the ground in a 37°C incubator. The upper esophagus was gently clamped with a plastic clip (to suspend the esophagus vertically), and then a button battery was inserted into the middle of the esophageal segment. Because the esophagus was elastic, the button battery would maintain the vertical position after been inserted into the esophagus.

In the second part of the experiment, to mimic the position of the esophagus during the supine position in humans, the esophageal segments were positioned with their long axis parallel to the ground. The esophageal segments were set in a culture dish containing a button battery.

In both parts of the study, the esophageal segments were randomly allotted to one of six groups (six segments were used for each group) as follows: (1) untreated (nothing injected), (2) lemon juice, (3) orange juice, (4) colza oil, (5) peanut oil, and (6) olive oil. According to previous studies (8), for each treatment, the liquid (5 mL) was injected into the esophageal segments every 5 min for 6 h.

### Detection of Button Battery Discharge Capacity, Residual Voltage, and Tissue pH

A voltmeter was used to measure the discharge voltage at the outer wall of the esophageal segment (which wrapped around the button battery) between the central points against each electrode as the extra-esophageal discharge voltage once an hour for 6 h (recorded in millivolts, mV). After 6 h, the button battery was removed from the esophageal segment, and the residual voltage of the battery was measured using a voltmeter (recorded in volts, V). The pH value of the esophageal segment (the area near the negative electrode of the battery) was measured with pH test paper once an hour for 6 h.

### Hematoxylin and Eosin (H&E) Staining

At the end of the treatment (6 h), esophageal tissues that were in contact with the negative electrode of the battery were cut out, fixed in 10% neutral formalin, paraffin sectioned, and stained with H&E. Histopathological changes were microphotographed under a light microscope. Index of esophageal injury was scored by a pathologist with experiences in the histological evaluation of esophageal preparations as described previously (12): 0, no obvious lesions; 1, lesions and inflammatory cell infiltration were observed in the mucosal layer; 2, lesions reached the submucosa layer, there was obvious patchy erosion, capillary dilation, as well as mucosal and submucosal neutral granulocyte infiltration; 3, lesions involved the muscular layer and a typical ulcer occurred. And four sections (one from each quadrant) were analyzed for each specimen. Briefly, the injured site of the esophagus was cut into 4 parts from the 3, 6, 9, and 12, o'clock to the center, then embedded and cut into 5 μm sections. The very middle section of each part was used for H&E staining.

### Statistical Methods

All of the statistical analyses were performed using SPSS 16.0 (SPSS Inc., Chicago, IL, USA) and GraphPad Prism 7 software (GraphPad Software, San Diego, CA, USA). Data were analyzed by one-way analysis of variance followed by the Newman–Keul's test (multiple comparisons). The results are presented as the mean ± standard error of the mean. A *P* < 0.05 indicated a statistical significance.

## Results

### Edible Oils Reduced Button Battery-Induced Esophageal Damage

**Figure 1:**
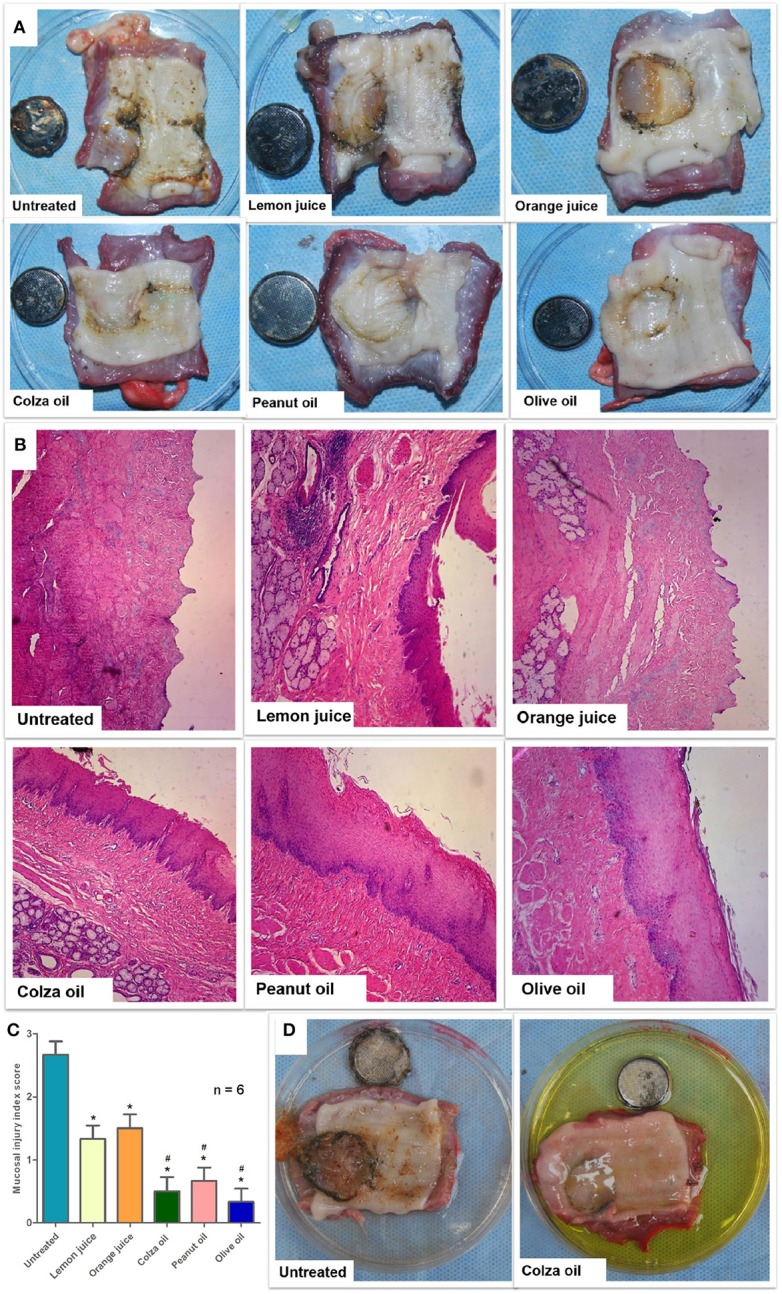
The effect of interventions on button-battery ingestion injuries (*n* = 6). **(A)** The gross esophageal damage induced by the button battery ingestion in the vertical position. **(B)** H&E staining of button battery-containing esophageal tissues treated with various edible oils (10×). **(C)** Mucosal injury index score according to H&E staining. Data were presented as mean with SEM. *P < 0.05 vs. untreated segments; ^#^*P* < 0.05 vs. lemon juice. **(D)** The effect of colza oil on button battery ingestion injuries in the supine model.

As shown in Figure 1A, the untreated battery-containing esophageal segments were severely damaged (the mucous membrane that was in contact with the negative electrode of the battery was eroded). The battery-containing esophageal segments treated with either lemon juice or orange juice were partially eroded. The battery-containing esophageal segments had little damage after treatment with either colza oil, peanut oil, or olive oil.

H&E staining was conducted to evaluate the histopathological changes induced by button battery contacted to the pig esophageal segments (Figure 1B). The mucosal and submucosal esophageal layers in the untreated esophageal segments were missing (eroded). The mucosal layer of the lemon juice- or orange juice-treated esophageal segments was either thin or absent (partially eroded), and the submucosal structure was destroyed. In contrast, the mucosal layer of the colza oil-, peanut oil-, or olive oil-treated esophageal segments was intact, and the structure of the submucosal membrane was intact. The mean mucosal injury index score (IIS, Figure 1C) was 2.7 ± 0.2 in the untreated esophageal segments and presented with mean ± standard error. The lemon juice-treated (1.3 ± 0.2, *P* = 0.002), orange juice-treated (1.5 ± 0.2, *P* = 0.008), colza oil-treated (0.5 ± 0.2, *P* < 0.001), peanut oil-treated (0.7 ± 0.2, *P* < 0.001), and olive oil-treated (0.3 ± 0.2, *P* < 0.001) segments had lower IISs when compared to the untreated segments. In addition, the colza oil-, peanut oil-, or olive oil-treated segments had lower IISs when compared to the lemon juice- (*P* = 0.021, *P* = 0.049, *P* = 0.028, respectively) or orange juice-treated segments (*P* = 0.028, *P* = 0.022, *P* = 0.007, respectively). No difference was observed in the IIS between the lemon juice- and orange juice-treated segments (*P* = 0.993). Also, no difference was observed in the IIS among the colza oil-, peanut oil-, and olive oil-treated segments (*P* = 0.993, *P* = 0.993, *P* = 0.879, respectively).

In the supine-positioned esophageal segments, esophageal injury was observed and photographed with the naked eye after the 6-h treatment (Figure 1D). The most serious esophageal injury was observed in the untreated segments, where the mucous membrane in contact with the negative electrode of the button battery was seriously damaged. The least esophageal injury was observed in the colza oil-treated segments, where the mucous membrane was basically intact.

### Edible Oils Reduced Button Battery Discharge in the Esophageal Segments

**Figure 2:**
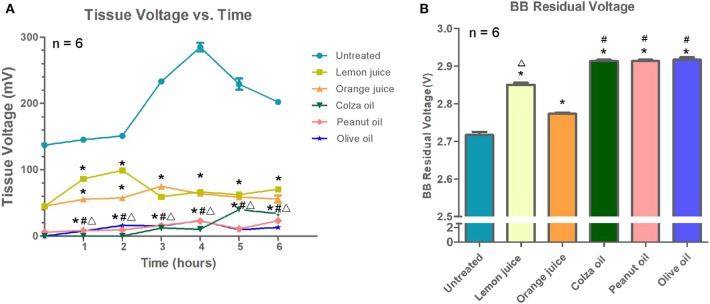
The effect of interventions on button battery discharge (*n* = 6). Data were presented as mean with SEM. **(A)** The extra-esophageal voltage discharge of inserted batteries. **(B)** The remaining voltage of the button battery of each intervention group after 6 h. *P < 0.05 vs. untreated segments; ^#^*P* < 0.05 vs. lemon juice; Δ*P* < 0.05, vs. orange juice.

As shown in Figure 2A, the highest extra-esophageal discharge voltage was observed in the untreated esophageal segments, while lower voltage levels were observed in the lemon juice- or orange juice-treated segments, and the lowest voltage levels were observed in the colza oil-, peanut oil-, or olive oil-treated segments (*P* < 0.001).

The button batteries were removed after 6 h of treatment, and the residual voltage of each button battery is shown in Figure 2B. Briefly, the residual voltage of the button batteries collected from the untreated segments (2.717 ± 0.0088 V) was lower than that of the button batteries collected from the lemon juice-, orange juice-, colza oil-, peanut oil-, or olive oil-treated segments (2.850 ± 0.0057 V, 2.773 ± 0.0033 V, 2.913 ± 0.0033 V, 2.910 ± 0.0033 V, and 2.917 ± 0.0066 V, respectively; all *P* < 0.001). The residual voltage of the button battery collected from the colza oil-, peanut oil-, or olive oil-treated segments was slightly higher than that of the lemon juice- or orange juice-treated segments (all *P* < 0.001). The residual voltage of the button battery collected from the lemon juice-treated segments was higher than that of the button battery collected from the orange juice-treated segments (*P* < 0.001). No difference was observed in the button battery residual voltage among the colza oil-, peanut oil-, and olive oil-treated segments (all *P* > 0.99).

### Edible Oils Reduced the pH Value of the Button Battery Anode in the Esophageal Segments

**Figure 3:**
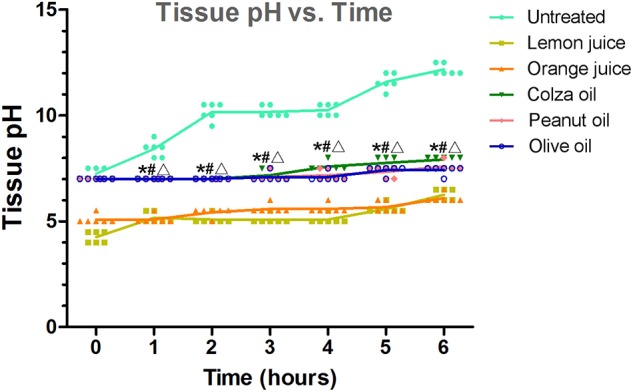
The effect of interventions on the pH value of the button battery anode in the esophageal segments. The tissue pH over time. Each data was staggered plotted. *P < 0.05 vs. untreated segments; ^#^*P* < 0.05 vs. lemon juice; Δ*P* < 0.05, vs. orange juice.

A standing model of a pig esophagus was used. As shown in Figure 3, in esophageal tissues that were in contact with the negative electrode of a button battery, more neutral pH values were observed in the colza oil-, peanut oil-, or olive oil-treated segments at each time point except 0 h compared to untreated segments with high pH (all *P* < 0.001), or compared to lemon juice- or orange juice-treated segments with lower pH (all *P* < 0.001).

## Discussion

Button batteries, commonly used in our daily life, can cause significant life-threatening damage to esophageal tissues after accidental ingestion. It is reported the button battery-induced damage can occur as early as 15 min after ingestion (8). However, there may be multiple delays from the time of ingestion until the patient arrives in a hospital for endoscopic removal of battery; this process often takes 6 h (9, 10). Thus, an early treatment strategy that would help to prevent or reduce persistent battery injury in esophagus before its removal is of particular scientific and clinical importance. In this study, from our *in vitro* experiment, we demonstrated that use of edible oils (i.e., colza oil, peanut oil, and olive oil) might be an effective way to minimize esophageal battery injury before removal.

The button battery-induced esophageal injury model was successfully developed in the current study. As expected, the insertion of a button battery into untreated pig esophageal segments caused severe damage. Interestingly, decreased levels of esophageal injuries were recorded when the button battery-containing esophageal segments were consistently (5 mL every 5 min for 6 h) injected with lemon juice or orange juice, indicating a quick protection strategy for patients who have ingested a button battery. Of particular note, we found that the button battery-induced esophageal injury could be basically prevented when the esophageal segments were consistently injected with colza oil, peanut oil, or olive oil. From these results, we believe that edible oils could be used as an alternative preoperative treatment or a supplemental therapy in case of that the removal of the ingested batteries couldn't be conducted in time, as fast removal is sure-enough the golden rule for its treatment. In addition, in our experiment, decreased levels of esophageal injury were observed in the segments perpendicular to the ground when compared to the segments parallel to the ground. It may seem that the standing position would be better than the supine position for patients who have ingested a button battery. However, the esophagus in the mediastinum does not hang freely as in the *in vitro* experiments, but is surrounded by tissue and compressed. There is also the esophageal peristalsis. Then, the location of the batteries in the esophagus and the pressure of the contacted mucosa were influenced by multiple factors. Therefore, the *in vivo* effect of patients' position on esophagus damage needs further verification.

The removal of the batteries from the esophageal lumen could be very difficult as the batteries adherent tightly to the mucosa as the duration increases (13). Preoperative treatment with oils may not only stop the electrolysis reaction, but also the batteries release from the mucosa may be facilitated. Because of esophageal peristalsis, this may also increase the likelihood that the batteries could directly pass through the esophagus and stomach, even without complications (14). But, as the battery becomes oily and slippery, it could be very difficult to grasp and recover the batteries with grasping forceps. Under such conditions, the retrieval baskets or nets may help to grasp the batteries (15). Otherwise, the batteries could be pushed into the stomach if couldn't be directly retrieved (14).

What are the protective mechanisms of edible oils, and why is edible oils more effective than lemon and orange juices? According to a recent study (8), button battery induces esophageal injury in two steps: (1) battery discharges within the esophageal electrolyte environment, and (2) creates a strongly alkaline environment in the esophagus. Battery discharge, which induces significant hydroxide accumulation around the negative electrode, is the major cause of esophageal injuries (8, 16). Higher discharge happens in a stronger electrolytic environment; lower discharge happens in a weaker electrolytic environment (e.g., acetic acid); while no discharge happens in a non-electrolytic environment (e.g., oil). Oil is a non-electrolyte that could isolate the battery from the electrolytic environment. When the battery is coated by oil, the battery discharge, and subsequently the formation of alkaline substances, greatly reduced or even stopped. Lemon juice and orange juice are weak electrolytes which can only reduce battery discharge. Although they have low acidic pH which can partially neutralize the alkaline substance produced by battery discharge, the battery will keep discharging to produce alkaline substances until surgical removal. Therefore, the juices can only partially reduce the damage of the esophageal mucosa.

The latest study conducted by Anfang et al. showed that honey or Carafate can attenuate button battery-induced esophageal injury (11). Their method has been written into the latest version of National Capital Poison Center Button Battery Ingestion Triage and Treatment Guideline (https://www.poison.org/battery/guideline). Some researchers are concerned that these highly viscous materials may lose their viscosity soon after consumption because of saliva-induced dilution (11). Since the edible oil is a fat-soluble substance while the saliva is a water-soluble solution, edible oil might not be easily dissolved and diluted in the saliva from the theoretical deduction of the “like dissolve like” rule. In addition, edible oils are non-electrolytic in physical nature, which will decrease the discharge of the battery, and in turn, reduce the production of alkaline. Hence, the use of edible oils may be a better option for the treatment of battery-induced esophageal injury.

However, what needs to be pointed out is that, compared to the tasty flavor of honey and sucralfate which were recommended in Anfang et al. (11), the taste of edible oil is not pleasant, and may cause resistance to oral administration in pediatric patients. As the palatability of edible oils could be attributed to several factors such as fat acid components and oxidation state (17, 18). It's possible that the flavor of edible oils could be modified and become more palatable to children. Another concern was the potential side effects of edible oil ingestion. Generally, the intake of edible oil will not cause aspiration when the patient was awake and alert as their pharyngeal reflex is normal. However, aspiration may occur if the patients' consciousness was compromised (19). Fortunately, the pulmonary aspiration is rare and has low morbidity and mortality in children under general anesthesia (19). The volume of 5 mL per 5 min for 6 h is a very large volume as compared to 3 mL/kg clear water 1 h before anesthesia (20). But the volume per hour equals to the previous study of Anfang et al. (11). The total volume difference is due to the different exposed durations of 2 h in Anfang's study vs. 6 h in present study. Although the stomach empties over time, the end volume in the stomach still needs to be very careful noticed and handled before anesthesia. For anesthesia for such children, a full stomach should be recognized and treated accordingly (21). Nevertheless, the appropriate volume of edible oils to be administered should be evaluated with future studies.

This is the first study to demonstrate the effective use of edible oils (i.e., colza oil, peanut oil, and olive oil) in maintaining a non-electrolytic environment in esophageal tissues that are in contact with the negative electrode of a button battery and attenuating button battery-induced esophageal injury. Since edible oils are easy to obtain and safe to drink, they should be considered during the pre-hospital care period for patients who have ingested a button battery. The results of the current *in vitro* study should be further investigated with regard to the smallest amount of oils needed to accomplish these results *in vitro* and then progress to clinical studies. The possibility of using edible oils in other anatomic areas such as the nasal cavity should be explored as well.

## Data Availability Statement

The datasets generated for this study are available on request to the corresponding author.

## Ethics Statement

The study was reviewed and approved by the institutional review committee at Air Force Medical University (No: 20180703).

## Author Contributions

WJ, WZ, and BZ conceived of the study. WJ, BZ, and QW designed experiments. GX and JX performed the pathology experiments. NS and WY performed the biophysical examinations. HW and QW analyzed data. WJ and WZ wrote the manuscript. All authors critically reviewed the manuscript.

### Conflict of Interest

The authors declare that the research was conducted in the absence of any commercial or financial relationships that could be construed as a potential conflict of interest.
